# Intravitreal Anti-Vascular Endothelial Growth Factor Therapies for Retinal Disorders

**DOI:** 10.3390/ph16081140

**Published:** 2023-08-11

**Authors:** Abraham Hang, Samuel Feldman, Aana P. Amin, Jorge A. Rivas Ochoa, Susanna S. Park

**Affiliations:** 1 Department of Ophthalmology & Vision Science, Ernest E. Tschannen Eye Institute, University of California Davis Eye Center, 4860 Y Street, Sacramento, CA 95817, USA; abhang@ucdavis.edu (A.H.); srfeldman@ucdavis.edu (S.F.); 2 School of Medicine, University of California Davis, Sacramento, CA 95817, USA; apamin@ucdavis.edu (A.P.A.); jarivasochoa@ucdavis.edu (J.A.R.O.)

**Keywords:** vascular endothelial growth factors (VEGFs), anti-VEGF therapy, intravitreal therapy, retinal disease, retinal neovascularization, choroidal neovascularization

## Abstract

Vascular endothelial growth factors (VEGFs) are key mediator of retinal and choroidal neovascularization as well as retinal vascular leakage leading to macular edema. As such, VEGF plays an important role in mediating visually significant complications associated with common retinal disorders such as diabetic retinopathy, retinal vein occlusion, and age-related macular degeneration. Various drugs that inhibit vascular endothelial growth factors (anti-VEGF therapies) have been developed to minimize vision loss associated with these disorders. These drugs are injected into the vitreous cavity in a clinic setting at regular intervals. This article provides an overview of the various anti-VEGF drugs used in ophthalmology and the common retinal conditions that benefit from this therapy.

## 1. Introduction

Vascular endothelial growth factors (VEGFs) are key mediators of retinal and choroidal neovascularization as well as vascular leakage, which can both contribute to vision loss associated with common retinal disorders. Neovascularization is a broad term that describes the formation of new blood vessels. Neovascularization is critical for normal development and maintenance of normal physiology, but derangement of this process leads to pathologic neovascularization. Pathologic neovascularization in the retina and choroid can occur secondary to a number of processes, including inflammatory, neoplastic, and ischemic etiologies. Vascular leakage in the retina occurs when there is a breakdown of the blood–retinal barrier. This can occur with retinal vasculopathy and pathologic neovascularization, contributing to vision loss.

It was long theorized that there were signaling factors mediating neovascularization. In 1939, Ide et al. postulated in a tumor animal model that there was a “blood vessel growth factor” [1]. However, it was not until 1983 that Harold Dvorak and colleagues demonstrated for the first time that a molecule they termed the vascular permeability factor was secreted by tumor cells [2]; blocking this molecule using an antibody could prevent vascular leakage. This molecule was later renamed to VEGF by Napoleone Ferrara, who isolated and cloned the molecule in 1989 and subsequently reported suppression of tumor growth in vivo using monoclonal antibodies to VEGF [2].

Since then, it has been found that there are several types of VEGF making up the VEGF family: VEGF-A, VEGF-B, VEGF-C, VEGF-D, VEGF-E (viral VEGF), VEGF-F (snake venom VEGF), placental growth factor (PlGF), and endocrine gland-derived VEGF (EG-VEGF) [3]. Furthermore, splicing results in different isoforms of VEGF. For example, VEGF-A_121_ and VEGF-A_165_ are two isoforms of VEGF-A comprised of different amino acid chain lengths denoted by the subscript [4]. VEGF-A is the most well-researched in the VEGF family. It has been strongly associated with angiogenesis and is induced by tissue hypoxia [5]. Specifically, VEGF-A_165_ has been the most extensively studied and may be the most predominant isotype [6].

VEGF molecules stimulate cellular responses by binding to specific receptors. These tyrosine kinase VEGF receptors (VEGFR) are divided into three main subtypes: VEGFR-1, VEGFR-2, and VEGFR-3. VEGFR-1 and VEGFR-2 are predominantly expressed in vascular endothelial cells, which also express the coreceptors neuropilin-1 (NP-1) and neuropilin-2 (NP-2), which modulate receptor function; VEGFR-3, in contrast, is expressed highly on lymphatic endothelial cells [3].

VEGF is a heparin-binding homodimeric glycoprotein [7] that plays an important role in both retinal physiology and pathology. VEGF is produced and secreted in a number of retinal cell types, including vascular endothelial cells, retinal pigment epithelial cells, muller cells, ganglion cells, and astrocytes [8]. VEGFR-1 and VEGFR-2 are expressed in the retina and have been identified in retinal vascular, glial, and neural cells [8].

In the retina, VEGF has multiple functions. It is involved in two different types of neovascularization: vasculogenesis (de novo blood vessel formation in development) and angiogenesis (blood vessel growth from existing vessels). VEGF also induces vascular permeability in pathologic states. VEGF may also have additional functions, including neuroprotection and retinal leukostasis [9,10,11,12,13,14,15]. In a murine model, VEGF-A expression was found to play an important role in the normal development of retinal and choroidal vasculature [16,17]. In humans, it is well established that VEGF-A is a key mediator of pathologic retinal and choroidal neovascularization and vascular leakage. Less is known about how extensively VEGF is involved in the routine maintenance of normal adult retinal vasculature and neuronal tissue. This is an important area of ongoing research since drugs that inhibit VEGF (anti-VEGF) are often injected into the vitreous cavity repeatedly and indefinitely for the management of certain retinal disorders.

In this review paper, we will discuss the common retinal diseases where VEGF overexpression is associated with pathogenesis and vision loss. We will also provide an overview of the pharmacologic agents that target VEGF and have been utilized to manage these conditions.

## 2. Retinal Disorders That Benefit from Anti-VEGF Therapies

### 2.1. Choroidal Neovascularization

#### 2.1.1. Exudative Age-Related Macular Degeneration

The most common retinal condition that ophthalmologists treat with anti-VEGF therapy is exudative age-related macular degeneration (AMD). AMD affects the macula, which is a small but critical area of the retina that is responsible for central vision. AMD has two stages, non-exudative (or “dry”) AMD and exudative (or “wet”) AMD.

**Figure 1:**
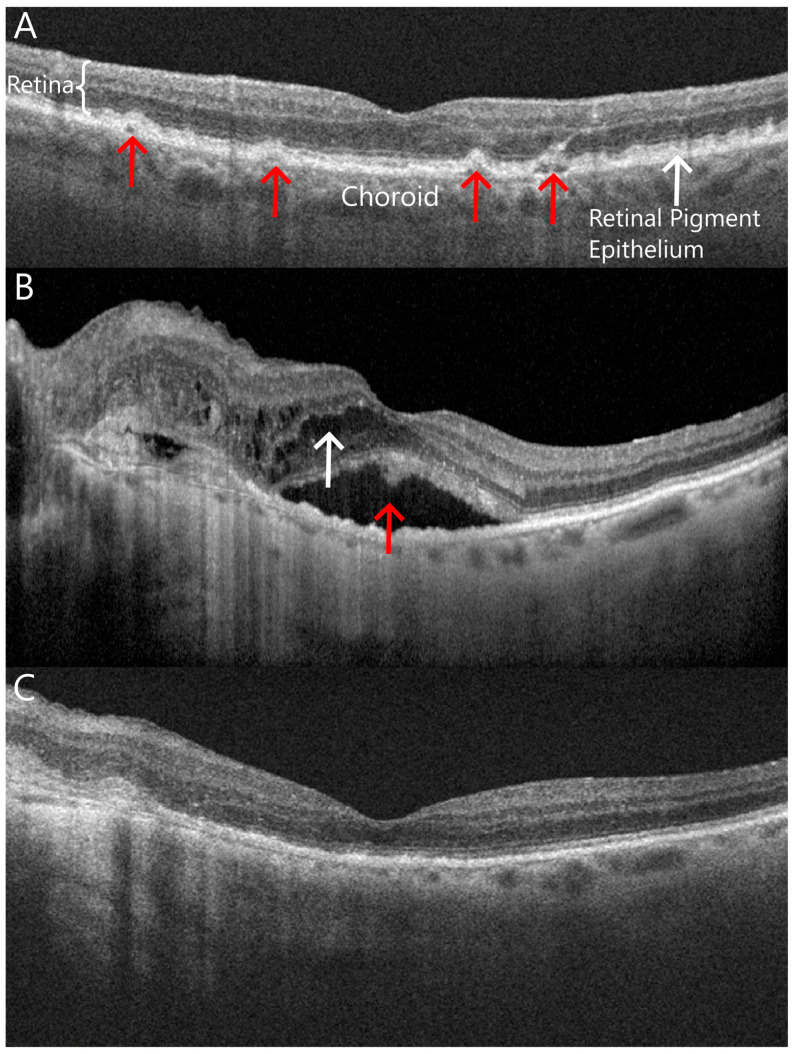
Optical coherence tomography images show cross-sectional in vivo images of the macula in the eyes in various stages of age-related macular degeneration (AMD). (**A**) An eye with non-exudative or “dry” AMD with drusen between Bruch’s membrane and the retinal pigment epithelium (red arrows); (**B**) an eye with active exudative or “wet” AMD with subretinal (red arrow) and intraretinal fluid (white arrow) in the macula; (**C**) the same eye shown in B after receiving several intravitreal anti-VEGF injections with interval resolution of intraretinal and subretinal fluid.

Non-exudative AMD is diagnosed clinically based on the presence of soft drusen in the macula. Drusen are collections of extracellular material that accumulate under the retina, between the retinal pigment epithelium (RPE) and Bruch’s membrane [18] (Figure 1A). The RPE has a critical role in maintaining the normal function of the retina [19]. The presence of soft drusen in the macula is an early clinical sign of RPE dysfunction, and the increasing number and size of soft drusen are early signs of progression of non-exudative AMD. In more advanced stages, degeneration of the photoreceptors, RPE, Bruch’s membrane, and choriocapillaris occurs, leading to significant vision loss.

Exudative or neovascular AMD develops when breaks in Bruch’s membrane lead to the growth of new vessels from the choriocapillaris into the subretinal space. The subretinal choroidal neovascular tissue subsequently causes subretinal bleeding or exudation and fluid in the macula (Figure 1B). The progression to exudative AMD is usually associated with new acute vision loss, which progresses without treatment. Intravitreal injections of anti-VEGF agents limit vision loss associated with exudative AMD by inhibiting the bleeding and exudation associated with choroidal neovascularization (Figure 1C). As such, intravitreal anti-VEGF treatment has become a mainstay in the treatment of exudative AMD.

#### 2.1.2. Polypoidal Choroidal Vasculopathy

In recent years, new imaging modalities have been developed to better visualize the choroid and retina in vivo. These new imaging tools have helped characterize pachychoroid neovasculopathy and polypoidal choroidal vasculopathy (PCV) as unique and separate entities from AMD [20]. These two conditions are characterized by a thickened choroid and dilated large choroidal vessels with obliteration of the choriocapillaris. The resulting choroidal ischemia and subretinal choroidal neovascularization are characteristic of pachychoroidal neovasculopathy and PCV [21]. Both conditions, in contrast to AMD, are more prevalent in younger, non-Caucasian populations [22].

In pachychoroid neovasculopathy and PCV, individuals can present with acute vision loss from serosanguineous detachments of the RPE in the macula and sub-retinal bleeding from choroidal neovascularization. Polypoidal dilations of the choroidal vessels can be seen with choroidal imaging in eyes with PCV. Intravitreal injections of anti-VEGF drugs can be used alone or in combination with verteporfin photodynamic therapy (PDT), depending on the anti-VEGF drug used. PDT involves intravenous infusion of a photosensitive medication followed by macular laser treatment to close the polyps and neovascular tissue in the choroid. One large study showed that PDT combined with the anti-VEGF drug, ranibizumab, was superior to ranibizumab alone in treating PCV [23,24]. A subsequent study showed that aflibercept, a newer anti-VEGF drug, was as effective when used alone to treat PCV as when combined with PDT [25,26,27].

#### 2.1.3. Other Retinal Entities Associated with Choroidal Neovascularization

Beyond exudative AMD and PCV, there are several other retinal conditions that can be associated with subretinal choroidal neovascularization and can benefit from anti-VEGF therapy. These include pathological myopia, presumed ocular histoplasmosis syndrome (POHS), posterior uveitis, angioid streaks, and trauma. Eyes with high myopia have elongated eyes in the anteroposterior axis and can develop secondary macular degeneration and subretinal choroidal neovascularization. The neovascularization is thought to result from mechanical chorioretinal stretching causing retinal and choroidal ischemia and the production of VEGF [28,29]. Eyes with POHS and posterior uveitis can develop chorioretinal scars and breaks in Bruch’s membrane in the macula that can lead to the development of subretinal choroidal neovascularization and vision loss. In eyes with angioid streaks, weakness in Bruch’s membrane leads to breaks in Bruch’s membrane, which can subsequently lead to vision loss from subretinal choroidal neovascularization. Angioid streaks can occur secondary to systemic conditions such as pseudoxanthoma elasticum, Paget’s disease of bone, and sickle cell disease. In patients with severe ocular injury, choroidal rupture with breaks in Bruch’s membrane can occur, which can induce the development of subretinal choroidal neovascularization. These rarer causes of choroidal neovascularization typically follow a more benign course than exudative AMD and often only require a limited course of anti-VEGF therapy [30].

### 2.2. Retinal Neovascularization

#### 2.2.1. Proliferative Diabetic Retinopathy

Diabetic retinopathy (DR) is the most common microvascular complication associated with diabetes mellitus. It is the most common retinal vasculopathy and the leading cause of blindness in working-aged adults in the developed world. Hyperglycemia leads to endothelial damage, capillary wall compromise, and the formation of retinal microaneurysms. Retinal microaneurysms are the earliest finding in DR and are the hallmark of non-proliferative diabetic retinopathy (NPDR). These microaneurysms can eventually rupture, forming dot-blot retinal hemorrhages seen on clinical eye exam [31]. Current standard therapy for NPDR focuses on achieving glycemic control to halt the progression of retinal ischemic damage and treating diabetic macular edema (DME) if present (see later Section 2.3).

**Figure 2:**
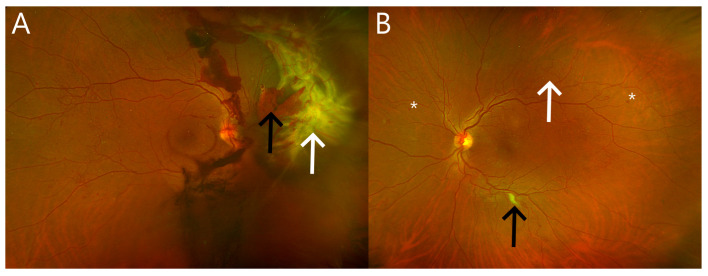
Wide field pseudocolor retinal images of eyes with proliferative diabetic retinopathy. (**A**) Right eye with florid neovascularization of the optic disc and retina resulting in tractional retinal detachment (white arrow) and hemorrhage in multiple layers including the vitreous (black arrow). (**B**) Left eye of the same patient showing less advanced proliferative diabetic retinopathy with less florid retinal neovascularization along the major retinal vessels (white arrow), a cotton wool spot (black arrow), and few dot-blot retinal hemorrhages (asterisks); no vitreous hemorrhage or a tractional membrane has occurred.

With sustained hyperglycemia, the number of microaneurysms and dot-blot retinal hemorrhages increases, causing retinal capillary occlusion and ischemia. Chronic retinal ischemia increases pro-angiogenic factors, including VEGF, which in turn drives retinal neovascularization. Retinal neovascularization is characterized by the growth of abnormal new vessels on the surface of the retina, unlike choroidal neovascularization, where the neovascularization is subretinal or sub-RPE in location. Once retinal neovascularization occurs, the disease is termed proliferative diabetic retinopathy (PDR). PDR is advanced DR and can be associated with severe vision loss from vitreous hemorrhage or tractional retinal detachment (Figure 2). Thus, prompt intervention is often indicated.

There are several options for the treatment of PDR. Traditionally, panretinal photocoagulation (PRP) has been used to reduce the angiogenic drive by thermally destroying the ischemic peripheral retina [32]. PRP provides a long-term reduction in angiogenic stimuli, making it a mainstay of treatment since its inception. Anti-VEGF drugs can be used as an alternative or adjuvant to PRP in treating PDR since it results in a rapid reduction of retinal neovascularization. There is a growing trend among retina specialists to use anti-VEGF therapy over PRP to treat PDR in patients with reliable follow-up and on chronic anti-VEGF therapy for concurrent diabetic macular edema (DME) [33]. Compared to PRP, chronic anti-VEGF therapy can preserve the peripheral vision in the short-term in eyes with PDR, although this benefit becomes insignificant with a longer follow-up of 5 years, according to the DRCR study, Protocol S [33]. The greatest drawback of using anti-VEGF monotherapy to treat PDR is the need for repeated injections, usually indefinitely, to sustain the treatment effect. Anti-VEGF therapy is also used in eyes with advanced PDR requiring surgery to reduce intraoperative and postoperative vitreous hemorrhage. It is also used to control PDR in eyes with significant vitreous hemorrhage with an inadequate view of the retina to deliver PRP.

Clinical trial data suggests that anti-VEGF therapy may have the ability to reduce the rate of progression of moderate and severe NPDR to PDR, but long-term data from DRCR Protocol W showed no visual benefit of this intervention [34,35]. Thus, the use of anti-VEGF in eyes with NPDR is limited to eyes with vision loss and center-involving DME—see Section 2.3.

#### 2.2.2. Retinal Vein Occlusion

Retinal vein occlusion (RVO) is the second most common retinal vasculopathy. RVO is classified based on the location of the vascular obstruction. In central retinal vein occlusion (CRVO), the obstruction occurs within the central retinal vein located within the optic nerve and affects the entire retina. In branch retinal vein occlusion (BRVO), the obstruction occurs within one of the branches of the retinal vein, usually at the site where the retinal artery and vein cross. Thus, only a segment of the retina is affected. Commonly identified risk factors for the development of RVO include increasing age, hypertension, diabetes mellitus, and open-angle glaucoma. Other possible risk factors for developing RVO include external compression, inflammation, coagulopathies, and vasospasm [36,37]. Long-standing vessel blockage can lead to varying degrees of retinal ischemia and macular edema (ME). The buildup of pressure within the obstructed retinal veins can cause damage to the vascular wall, leading to vascular leakage and the formation of ME. The sudden transient retinal ischemia induces the release of VEGF and other inflammatory factors, which also contribute to the development of ME [38]. ME associated with RVO results in further reduction in vision and requires treatment. The first intravitreal therapy used to treat ME associated with RVO was triamcinolone acetonide. Intravitreal corticosteroids are highly effective and are still used today for treating ME [39]. However, it is no longer the first-line treatment since intravitreal corticosteroids can cause glaucoma and cataract. Intravitreal anti-VEGF therapy is currently the first-line treatment of ME associated with RVO, as it has been shown in large studies to preserve visual acuity compared to other therapies—see Section 2.3 [40,41].

RVO associated with a greater degree of retinal ischemia may develop retinal or iris neovascularization. Once ocular neovascularization occurs, retinal laser photocoagulation is delivered to the ischemic retina to decrease the long-term production of VEGF. As in PDR, anti-VEGF therapy can be used for prompt control of ocular neovascularization, but it does not provide long-term control.

#### 2.2.3. Iris Neovascularization/Neovascular Glaucoma

Neovascular glaucoma is a severe and potentially blinding complication associated with both PDR and RVO. Ocular ischemia increases VEGF production and leads to the formation of new blood vessels in the iris and anterior chamber. New abnormal blood vessels first begin to grow over the iris and then extend to the peripheral iris and trabecular meshwork. The latter results in obstruction of aqueous humor outflow through the trabecular meshwork, which increases intraocular pressure (IOP). The rise in IOP is often severe and acute and can result in severe, permanent vision loss if not treated promptly.

The current mainstay of treatment of iris neovascularization and neovascular glaucoma is anti-VEGF therapy and PRP [42]. Anti-VEGF therapy is often useful in this condition due to its ability to regress the neovascular membranes more quickly than PRP. PRP is often used for long-term control of neovascularization. The combination of intravitreal ant-VEGF therapy and PRP leads to rapid and long-term reduction of angiogenic stimuli and regression of neovascular vessels. Thus, they are often used together to provide the most optimal outcome. However, IOP can remain elevated even after regression of neovascularization. Such eyes require incisional glaucoma surgery or ablation of the ciliary body to minimize the risk of further vision loss from glaucoma.

#### 2.2.4. Retinopathy of Prematurity

Retinopathy of prematurity is a leading cause of blindness worldwide in extremely premature infants [43] and is postulated to be the result of many factors. Continued improvement in neonatal care has resulted in premature neonates surviving at increasingly younger gestational ages [43]. The resulting underdevelopment and increased oxygen demand imposed on preterm infants lead to hyperoxygenation, which damages existing retinal vessels and creates a paradoxical avascular environment with a new demand for angiogenesis [44]. The rapid influx of angiogenic stimuli creates aberrant retinal neovascularization with vessels growing into the vitreous gel instead of the retina. If this abnormal retinal neovascularization is not treated promptly, the resulting fibrovascular tissue can cause tractional retinal detachment and vision loss. Permanent vision loss and blindness from retinal detachment can occur even after surgical intervention.

Traditionally, retinal laser photocoagulation of the ischemic peripheral retina has been used to control retinal neovascularization and minimize the risk of vision loss associated with the progression of retinopathy of prematurity [45]. However, retinal laser photocoagulation is less effective in minimizing the progression of retinopathy of prematurity if neovascularization involves the more posterior retina, i.e., zone 1 disease. Furthermore, in these eyes, laser treatment can be associated with permanent loss of peripheral vision, which, in turn, may contribute to the development of severe myopia. For these eyes with zone 1 disease, intravitreal anti-VEGF has been shown to be more effective than laser treatment and is associated with progressive maturation of the retinal vessels [45]. The use of intravitreal anti-VEGF treatment for retinopathy of prematurity has been steadily increasing [46] since it is much easier to administer than laser photocoagulation, results in a rapid reduction of angiogenic drive and retinal neovascularization, and allows for further maturation of the peripheral retinal vasculature. However, anti-VEGF treatment may not be permanent and late reactivation of retinal neovascularization can occur following anti-VEGF therapy. Thus, eyes with retinopathy of prematurity treated with anti-VEGF therapy require close, prolonged follow-up with frequent screening eye examinations. In addition, since there are limited data regarding the long-term safety of intravitreal anti-VEGF therapy in neonates, there are on-going concerns regarding potential long-term systemic risks of anti-VEGF therapy in the development of neonates.

### 2.3. Macular Edema

#### 2.3.1. Diabetic Macular Edema

Diabetic macular edema (DME) is the most common indication for anti-VEGF treatment in eyes with DR. DME can present in the early or later stages of DR and can result in vision loss. Chronic hyperglycemia causes loss of pericytes that are normally present on capillary walls. This change causes retinal microaneurysm formation and capillary leakage. Additionally, there is an inflammatory component in the development of DME, with significant upregulation of VEGF and other chemokines and cytokines [47]. These inflammatory factors increase vascular permeability, further contributing to the development of DME. Prior to the development of intravitreal anti-VEGF therapies, laser photocoagulation was used to treat microaneurysms and areas of retinal thickening in the macula. Multiple clinical trials have shown the superiority of intravitreal anti-VEGF therapy over macular laser treatment in preserving vision in eyes with DME [48,49]. Thus, intravitreal anti-VEGF therapy is now the standard of care for treating this condition.

#### 2.3.2. Retinal Vein Occlusion

As discussed in an earlier section, RVO occurs when a thrombus occludes a retinal vein, and this can occur at the level of the central retinal vein or at a branch retinal vein (known as CRVO and BRVO, respectively). One of the most common complications of RVO is ME. ME occurs in this condition due to the release of VEGF and other inflammatory factors from the hypoxic retina and can contribute to vision loss [50]. Intravitreal anti-VEGF therapy has transformed the treatment of RVO, and many studies have shown their efficacy and superiority over macular laser photocoagulation in preserving vision [51,52,53].

#### 2.3.3. Radiation Retinopathy

Radiation retinopathy can develop in the eyes following head and neck radiotherapy, typically one to three years after radiation exposure. Clinically, radiation retinopathy looks very similar to DR and is more severe in patients with DR. Radiation exposure can cause endothelial cell loss, occlusion of capillary beds, and microaneurysm formation [54]. Retinal ischemia develops, leading to VEGF and inflammatory cytokine upregulation, which in turn causes ME. Intravitreal anti-VEGF therapy has been an effective tool for treating ME related to radiation retinopathy, though macular ischemia often limits visual potential [55].

### 2.4. Other Indications

#### 2.4.1. Retinal Artery Macroaneurysm

Retinal artery macroaneurysm is a focal dilation of a branch of the retinal artery that can be associated with vision loss if complicated by retinal hemorrhage or ME [56]. This condition occurs predominantly in females and is associated with chronic hypertension and atherosclerotic disease. The condition is diagnosed primarily by clinical exam. There are no definitive guidelines for treating retinal artery macroaneurysm since they often resolve spontaneously. Intravitreal anti-VEGF therapy can be useful to treat associated ME, if present, to minimize vision loss [57]. In certain cases, laser photocoagulation can be considered, although this is controversial as there is a risk of inducing sectoral retinal ischemia.

#### 2.4.2. Peripheral Exudative Hemorrhagic Chorioretinopathy

Peripheral exudative hemorrhagic chorioretinopathy, as the name suggests, describes a condition that is associated with hemorrhage and exudation of the peripheral retina, usually from subretinal choroidal neovascularization. This condition is usually seen in elderly individuals and is more common in Caucasians and females. Associated risk factors include hypertension, AMD, and anticoagulation use. This condition is usually asymptomatic and has a self-limiting course. Occasionally, associated exudation and hemorrhage may be severe and encroach on the macula. In such eyes, intravitreal anti-VEGF therapy can be considered to minimize the risk of vision loss [58]. There are insufficient data to suggest a clear guideline for treating this condition as it is an uncommon diagnosis.

## 3. Intravitreal Anti-VEGF Therapies for Retinal Disorders

### 3.1. Pegaptanib

Pegaptanib (Macugen) was developed by Eyetech Pharmaceuticals, Inc. (New York City, NY, USA) and received FDA approval in December 2004 as the first aptamer therapeutic in humans. It was also the first intravitreal anti-VEGF drug to receive FDA approval to treat retinal disorders. It is a 28-base ribonucleic acid aptamer that binds with a high affinity to the heparin-binding domain of VEGF-A_165_ and inhibits signal transduction for vascular proliferation by disrupting VEGF-A_165_ interaction with VEGFR-1 and VEGFR-2 [7]. This drug was developed since VEGF-A_165_ was believed to be the major pathologic isoform of VEGF-A in the eye [6].

The phase 2 trials demonstrated that treatment with pegaptanib was superior to PDT in eyes with exudative AMD [59,60]. At the time of the clinical trial, PDT was the mainstay for treating exudative AMD. The study showed that 25% of patients on pegaptanib demonstrated an improvement of 3 lines or greater in visual acuity, compared to 2.2% of patients treated with PDT. Subsequent phase 3 trials demonstrated a dose-independent response to pegaptanib in eyes with exudative AMD, with low doses of 0.3 mg and 1 mg being just as effective in reducing neovascularization and preserving visual acuity as higher doses [61]. Adverse effects of the drug were noted, although the incidence was rare. The most serious adverse effects included endophthalmitis (<1.5%) and retinal detachment (<1%); other less serious side effects, including vitreous floaters, anterior chamber inflammation, corneal edema, and blurred vision, were also noted in post-marketing reports [62].

Pegaptanib, while an improvement over PDT, was a disappointing therapy for exudative AMD since the improvement in visual outcome was relatively low. The limited efficacy of pegaptanib was thought to be due to the binding of the drug to the heparin-binding domain of VEGF-A instead of the receptor-binding domain targeted by later anti-VEGF agents [63], as well as its limited activity targeting only one isoform of VEGF-A. By the early 2010s, pegaptanib was largely replaced by more effective anti-VEGF agents that inhibited all isoforms of VEGF-A.

### 3.2. Bevacizumab

Bevacizumab (Avastin) is a humanized monoclonal full-length recombinant IgG antibody that binds all VEGF-A isoforms with two binding sites per molecule. It was approved by the FDA for intravenous use against colorectal cancer in 2004 [64,65]. As ophthalmologists were transitioning to using pegaptanib to treat exudative AMD, promising phase 3 trial data of ranibizumab for the treatment of exudative AMD were being reported. However, the FDA had not yet approved intravitreal ranibizumab, a monoclonal antibody fragment derived from the same parent antibody as bevacizumab. Since patients with exudative AMD were continuing to lose vision using pegaptanib, ophthalmologists began experimenting with off-label use of bevacizumab, an antibody with the same parent molecule as ranibizumab which was already available on the market for the treatment of colon cancer [63,66,67,68]. An initial pilot study was done using intravenous bevacizumab, which demonstrated improved visual acuity and retinal thickness in eyes with exudative AMD [66]. A couple of case reports appeared in the literature in 2005, demonstrating that intravitreal bevacizumab was effective and well-tolerated in eyes with exudative AMD [68] and ME from CRVO [67]. These case reports ultimately paved the way for further studies exploring the off-label use of intravitreal bevacizumab in the treatment of many different retinal pathologies [69,70].

Today, intravitreal bevacizumab remains a very popular option due to its comparable efficacy and much lower cost when compared to on-label intravitreal anti-VEGF drugs. A multi-center clinical trial conducted comparing the efficacy of intravitreal bevacizumab to on-label ranibizumab for the treatment of exudative AMD found the two drugs to be comparable [71]. Currently, intravitreal bevacizumab is used off-label for a variety of retinal conditions associated with choroidal or retinal neovascularization or ME, including exudative AMD, choroidal neovascularization from pathologic myopia, PDR, DME, ME due to RVO, retinopathy of prematurity, neovascular glaucoma, and others [72]. The typical dose of intravitreal bevacizumab is 1.25 mg per 0.05 mL. Some studies on retinopathy of prematurity in neonates suggest that lower doses may provide equal efficacy and carry the potential to decrease cost and potential systemic adverse effects; other studies highlight that doses up to 4.0 mg may be beneficial for more resistant neovascularization [73,74]. Generally, the medication is well tolerated, and events such as endophthalmitis and retinal detachment are very rare despite the drug being aliquoted for intravenous injection by compounding pharmacies [75,76,77].

### 3.3. Ranibizumab

Ranibizumab (Lucentis), as mentioned earlier, is a monoclonal antibody fragment derived from the same parent monoclonal antibody as bevacizumab. It was developed by Genentech (South San Francisco, CA, USA) for intravitreal administration. Before intravitreal bevacizumab was used off-label clinically, researchers believed bevacizumab would be too large a molecule to penetrate into the subretinal space to reach the choroidal neovascular tissue in eyes with exudative AMD [78]. Thus, ranibizumab was developed as a small, 48 kD antibody fragment that binds to the receptor-binding domain of VEGF_121,_ VEGF_165_, and VEGF_189_ [79]. It also lacks an Fc region, allowing it to bypass Fc recycling and prolong its existence in the eye [80]. Ranibizumab received FDA approval in 2006 for the treatment of exudative AMD after two landmark phase III trials, MARINA and ANCHOR, showing superior visual acuity with limited adverse effects in eyes treated with monthly intravitreal ranibizumab (0.3 mg or 0.5 mg) when compared to the standard of care at the time, i.e., observation or PDT [81,82]. The MARINA trial at 12 months showed that almost 95% of eyes showed stable visual acuity, i.e., lost fewer than 15 letters; visual acuity improved by 15 or more letters in 24.8% of the 0.3 mg group and 33.8% in the 0.5 mg group (verses 5.0% in the sham group) [81]. Multiple studies since then have demonstrated the safety and efficacy of ranibizumab [83]. Currently, it is also FDA-approved for the treatment of PDR, DME, ME from RVO, and choroidal neovascularization due to myopia [84].

### 3.4. Aflibercept

Aflibercept (Eylea) was developed by Regeneron Pharmaceuticals, Inc. (Tarrytown, NY, USA) with the intention of creating a therapeutic for exudative AMD with sustained inhibitory effects against VEGF [85]. It was developed as a recombinant fusion protein comprised of the VEGFR-1 and VEGFR-2 cell surface receptor binding domains fused with an Fc portion of human immunoglobulin G [86]. Aflibercept’s structure allows it to function as a decoy receptor that binds VEGF-A with a high affinity, preventing it from interacting with its natural receptors and subsequently inhibiting angiogenic potentiation. This mechanism of action gives rise to aflibercept’s other name—VEGF Trap [86,87]. In addition to blocking the action of all isoforms of VEGF-A, aflibercept also binds to VEGF-B and is the only anti-VEGF agent in its class that inhibits placental growth factor (PlGF), another key modulator of neovascularization [87,88]. Therapeutic effects observed in two phase III clinical trials, VIEW1 and VIEW2 trials, led to the FDA approval of aflibercept for the treatment of exudative AMD in 2011. These trials investigated the efficacy of aflibercept 2 mg given bimonthly following an initial three doses given monthly; results showed that aflibercept effectively improved visual acuity and reduced retinal fluid and was non-inferior to ranibizumab given monthly [89,90]. Since aflibercept required fewer doses than ranibizumab as per the study design, the results suggest it may have a longer duration of action than ranibizumab. Aflibercept has since been FDA-approved for the treatment of other neovascular conditions, including DME [91], ME due to RVO [92], PDR, and choroidal neovascularization due to myopia [85].

A 2-year clinical trial comparing bevacizumab, ranibizumab, and aflibercept for DME found that all three drugs decreased DME and improved visual acuity after 2 years [93]. However, visual acuity with aflibercept was better than bevacizumab at 2 years in eyes with worse baseline visual acuity (20/50 or worse), suggesting that aflibercept may be superior in these cases. Visual acuity with aflibercept was better than ranibizumab at 1 year in eyes with worse baseline visual acuity, but this difference was not significant at 2 years.

Recent studies have compared the standard dosing of aflibercept of 2 mg versus a higher dose of 8 mg in patients with exudative AMD. The phase II CANDELA clinical trial demonstrated a potential trend in improved anatomic and visual outcomes with the higher dose aflibercept without any difference in safety profiles, although the primary endpoint did not achieve statistical significance [94]. Serious adverse effects occurred in less than 0.1% of patients receiving intravitreal injections of aflibercept. They include endophthalmitis, retinal detachment, and elevated intraocular pressure [95].

### 3.5. Brolucizumab

Brolucizumab (Beovu) is a single-chain variable antibody fragment with an absent Fc portion that binds all isoforms of VEGF-A. Intravitreal brolucizumab received FDA approval for the treatment of exudative AMD in the United States in October 2019 [96]. Originally developed by ESBATech (Schlieren, Zurich, Switzerland), brolucizumab contains a human single-chain variable fragment scaffold grafted to a complementarity-determining region of an anti-VEGF antibody, leading to a smaller molecule size [97]. This smaller molecular size was postulated to lead to higher tissue penetrance (2.2× higher exposure to the retina and 1.7× higher exposure to the RPE/Choroid complex compared to ranibizumab in preclinical data) [98]. It also allowed for a higher molar concentration of the drug in the 50 μL intravitreal injection, a >10-fold increase compared to aflibercept and a 20-fold increase compared to ranibizumab [99]. Two phase III clinical trials, HAWK and HARRIER, compared intravitreal brolucizumab to intravitreal aflibercept for treatment-naïve eyes with exudative AMD [98,100]. The studies demonstrated the non-inferiority of brolucizumab compared to aflibercept every 8 weeks in terms of visual acuity gain and anatomic resolution of retinal fluid. Since over 50% of eyes treated with brolucizumab were maintained on every 12-week dosing, the data suggested that brolucizumab may be longer lasting than aflibercept [98,100]. Brolucizumab alone, or in combination with aflibercept, has also been shown to be effective in treating patients with chronic exudative AMD resistant to other anti-VEGF therapies [101]. The KESTREL and KITE phase III clinical trials also demonstrated that brolucizumab was non-inferior to aflibercept in visual outcome in treating DME [102]. Unfortunately, after FDA approval, a number of post-marketing reports of severe vision loss due to intraocular inflammation (IOI) including retinal vasculitis and/or retinal artery occlusion were reported after treatment with brolucizumab [103,104]. A post hoc review of the HAWK and HARRIER studies showed an IOI incidence of 4.6% on brolucizumab (versus 1.5% on aflibercept), but incidence of severe vision loss was comparable in the two groups [105]. While brolucizumab remains an anti-VEGF treatment option for exudative AMD, the risk of IOI has limited its use in recent years. Since it is unclear at this time which eyes are predisposed to IOI, careful observation is necessary to monitor for any signs of inflammation in eyes undergoing treatment with brolucizumab.

### 3.6. Faricimab

Developed by Roche/Genentech (South San Francisco, CA, USA), Faricimab (Vabysmo) received approval from the FDA in January 2022 for the treatment of exudative AMD and DME in the United States [106]. Faricimab is a bispecific antibody inhibiting both VEGF-A and Angiopoetin-2 (Ang-2), another angiogenic growth factor. Ang-2 has been implicated in decreasing vascular integrity, having a complementary or perhaps synergistic effect with VEGF-A [106,107]. Faricimab has demonstrated non-inferiority in visual acuity outcome to aflibercept in the TENAYA and LUCERNE trials, two phase III clinical trials for exudative AMD [108], and YOSEMITE and RHINE trials, two phase III trials for DME [109]. Rates of ocular adverse events were comparable to aflibercept. Over 75% of eyes treated with faricimab could be maintained at an every 12 to 16 weeks treatment regimen, suggestive of prolonged durability of faricimab when compared to aflibercept. As the newest FDA-approved anti-VEGF therapy at this time, research is still ongoing regarding both the efficacy and safety profile of this medication.

## 4. Discussion

There is a growing number of anti-VEGF drugs that can be administered intravitreally to treat various common retinal disorders such as exudative AMD, PDR, and ME due to RVO or DR. The currently available agents mentioned in Section 3 are summarized in Table 1. Intravitreal administration of these drugs allows for high bioavailability of the drug in the retina for maximum therapeutic effect while minimizing the potential systemic side-effect of anti-VEGF therapy. In fact, intravitreal anti-VEGF therapies are highly effective in reducing ocular neovascularization, macular edema, and exudation that lead to vision loss.

Unfortunately, the anti-VEGF effects are reversible, and frequent repeat intravitreal injection of the drug is needed to maintain the therapeutic effect. This need for retreatment is often indefinite. Some eyes may become resistant to anti-VEGF therapy and require prolonged dosing as frequently as every 4 weeks. This retreatment regimen poses a high financial and social burden on patients. On-label intravitreal anti-VEGF drugs are expensive, and insurance companies often impose the use of off-label bevacizumab as a first-line treatment to limit drug costs. The majority of patients receiving intravitreal anti-VEGF therapy are elderly and visually impaired, and many need to rely on family and friends to provide transportation for these eye treatment visits.

Finally, intravitreal anti-VEGF injections can be associated with adverse events. Serious ocular adverse events, which can be associated with the injection itself, such as infection, bleeding, retinal detachment, and intraocular inflammation, can result in severe vision loss. Fortunately, these events are relatively rare. The more common patient complaints after intravitreal injections are eye irritation and blurry vision, which are experienced by almost all patients soon after injection. Eye irritation can persist for a couple of days after the injection. Systemic adverse events following intravitreal anti-VEGF are a concern, given the known adverse effects of hypertension, bleeding, and cardiovascular events associated with systemic anti-VEGF therapy. However, the incidence appears to be low and of reduced severity with intravitreal anti-VEGF therapy, given the very low dose of the drug administered. Intravitreal anti-VEGF agents have been associated with a short-term elevation in blood pressure after injection [110], although this effect may vary by anti-VEGF agent [111] and it is debatable whether it is of clinical significance. A systematic review and meta-analysis of over 9000 patients found no difference in rate of major cardiovascular and non-ocular hemorrhagic events in patients who received intravitreal ranibizumab or bevacizumab when compared to patients who did not receive anti-VEGF treatment, but this study may be underpowered to assess for rare adverse events [112].

## 5. Future Directions: Emerging Anti-VEGF Therapies

Included in this section are some of the new emerging strategies for intravitreal anti-VEGF therapy that are under development to reduce the cost and retreatment burden while maximizing efficacy.

### 5.1. Port Delivery System

In October 2021, the FDA approved Susvimo, a port delivery system (PDS) developed by Genentech (South San Francisco, CA, USA), for the treatment of exudative AMD in eyes that had previously received at least two anti-VEGF injections [113]. The PDS is a surgically delivered permanent intraocular implant that is refillable and designed to deliver ranibizumab continuously into the vitreous. The refill-exchange procedure is done in a clinic at 24 weeks intervals [113]. The implant is inserted surgically via a scleral incision into the vitreous cavity at the level of the pars plana and covered by conjunctiva; it can be refilled through a self-sealing septum. The Archway Phase 3 trial demonstrated that PDS every 24 weeks was non-inferior compared to monthly injections of ranibizumab for eyes with exudative AMD; 98.4% of eyes did not receive any supplemental treatment [113]. Adverse events included vitreous hemorrhage (5.2%), retinal detachment (0.8%), and endophthalmitis (1.6%). The non-inferiority of PDS to monthly ranibizumab has been demonstrated for up to 2 years with the most recently published Archway trial data [114].

The PDS was recalled by Roche/Genentech in October 2022 after reports of septum dislodgement within the implant after refill-exchange procedures. There were 33 cases reported in 1419 patients (2.3%) over 5236 refill-exchange procedures, and the PDS remains off the market at this time while quality issues are being resolved by the manufacturer [115].

### 5.2. Anti-VEGF Biosimilars

As some of the earlier anti-VEGF therapies have been on the market for over a decade now, their patents are expiring, leading the way for the development of anti-VEGF biosimilars. A biosimilar agent must have no meaningful difference in safety, efficacy, or potency compared to the reference drug. For ophthalmic use, they must also demonstrate safety and efficacy in the eye, which can be entirely different from systemic pharmacokinetics and pharmacodynamics. For this reason, the Academy of Ophthalmology strongly recommends against off-label use of biosimilars unless there is strong clinical data that the drug is safe and effective in the eye [116]. One example of this was the development of Razumab in India by Intas Pharmaceuticals, the first commercially available biosimilar to ranibizumab approved in India in 2015; Razumab was shown to have increased rates of sterile endophthalmitis, which eventually led to a recall and changes in drug formulation [117,118].

In the United States, there are currently two anti-VEGF biosimilars FDA-approved for ophthalmic use: ranibizumab-nuna (Byooviz; Biogen—Cambridge, MA, USA and Samsung Bioepis—Incheon, South Korea), which was FDA-approved in 2021 for exudative AMD, ME due to RVO, and myopic choroidal neovascularization, and ranibizumab-eqrn (Cimerli; Coherus—Redwood City, CA, USA), which was FDA-approved in 2022 as the first interchangeable biosimilar to ranibizumab [119]. There are currently numerous biosimilars under clinical trial for ophthalmic use. Ranibizumab biosimilars include Xlucane (Xbrane Biopharma—Solna, Sweden, Bausch + Lomb—Ontario, Canada, and Stada Arzneimittel—Bad Vilbel, Germany) [120]. Aflibercept biosimilars include MYL-1701P (Mylan—Canonsburg, PA, USA and Momenta—Cambridge, MA, USA), ABP 938 (Amgen —Thousand Oaks, CA, USA), FYB203 (Bioeq—Zug, Switzerland), SOK583A1 (Sandoz—Basel, Switzerland), SB15 (Samsung Bioepis—Incheon, South Korea), and CT-P42 (Celltrion—Incheon, South Korea) [119]. Two bevacizumab biosimilars are currently approved in the United States for non-ophthalmic oncologic indications only: bevacizumab-bvzr (Zirabev; Pfizer—New York, NY, USA) and bevacizumab-awwb (Mvasi; Amgen—Thousand Oaks, CA, USA) [121]. An ophthalmic bevacizumab biosimilar ONS-5010 (Outlook Therapeutics—Iselin, NJ, USA) is currently under investigation [121,122]. In the upcoming years, there will likely be an increasing number of biosimilars available on the market, which could lead to potential financial savings for patients and the healthcare system. However, increased vigilance post-marketing will be important to identify any possible adverse events, such as those seen with Razumab.

### 5.3. DARPins

Designed ankyrin repeat proteins (DARPins) are a relatively new class of non-immunoglobulin small molecule single-domain proteins that can bind to a target protein with very high specificity and affinity [123]. Abicipar pegol, developed by Allergan (Dublin, Ireland), is an anti-VEGF DARPin molecule. The phase III clinical trial data from the SEQUOIA and CEDAR studies demonstrated that over 90% of patients with exudative AMD maintained stable vision at 12-week dosing intervals, similar to monthly ranibizumab injections; however, rates of IOI were around 15% [124,125,126]. Although this rate was reduced to 8.9% in the subsequent MAPLE study after a modified manufacturing process [127], the rates of IOI were still much higher than the current anti-VEGF therapies on the market. Thus, the FDA declined the approval of abicipar pegol for exudative AMD in 2020.

### 5.4. Thermosensitive Hydrogels

Thermosensitive hydrogels are hydrophilic polymers that are liquid at room temperature but solid at body temperature. This unique property can allow drugs to be delivered into the eye while allowing for sustained release inside the eye [128,129]. These hydrogels are still being investigated in vitro and in animal models. Thomasy et al. demonstrated that aflibercept loaded into a “microsphere thermo-responsive hydrogel” could be injected intravitreally in rhesus macaques with sustained release of aflibercept for up to 6 months [130].

### 5.5. Tyrosine Kinase Inhibitors

GB-102, developed by Graybug Vision (Redwood City, CA, USA), is a biodegradable microparticle depot formulation of sunitinib malate [131], a small molecule receptor tyrosine kinase (RTK) inhibitor that is currently used to treat malignancies, including GI stromal tumors and advanced renal cell carcinoma [132]. The inhibition of receptor tyrosine kinase blocks both VEGF-A and platelet-derived growth factor activity, and the depot formulation of GB-102 can last up to 6 months in the eye [133]. Data from the Phase 2b ALTISSIMO trial showed that the frequency of injections was reduced by 58% compared to patients’ pre-trial treatment regimen [134].

### 5.6. Adjuvant Radiotherapy

Radiotherapy as an adjuvant to intravitreal anti-VEGF therapy has been explored in clinical trials to reduce the retreatment burden in eyes with exudative AMD. The rationale for exploring this combination therapy is based on observations made in oncology studies where sustained tumor regression with anti-VEGF therapy was only achieved when combined with radiotherapy or chemotherapy [135]. Radiation theoretically can reduce neovascularization by destroying endothelial cells. Radiotherapy to the macula has been applied using intravitreal brachytherapy, external beam radiotherapy via robotics, and proton beam therapy. Our group conducted a Phase I/II randomized, sham-controlled, double-masked study demonstrating that the combination of intravitreal anti-VEGF therapy and low-dose proton beam radiotherapy is well tolerated and associated with a significantly lower need for retreatment with intravitreal anti-VEGF in eyes with newly diagnosed exudative AMD [135,136]. However, on long-term follow-up of 4 years, the development of geographic atrophy limited visual acuity in some AMD eyes and was more common in eyes that received higher doses of radiation. Whether this observation is related to the long-term effects of radiation or from a natural progression of AMD could not be determined in our small study since geographic atrophy was more common at baseline in the eyes that received higher doses of radiation [136]. A similar reduction in anti-VEGF retreatment was noted in a multi-center, prospective, open-labeled study using epi-macular brachytherapy, but the visual outcome was compromised in the combination therapy group when compared to the anti-VEGF monotherapy group after 2 years [137]. In this study, it was unclear whether the reduced vision in the brachytherapy group was due to cataract progression from vitrectomy used for brachytherapy, the effect of radiotherapy, or the open-labeled study design potentially leading to undertreatment with anti-VEGF therapy. Thus, further research is needed to further assess the safety and efficacy of adjuvant radiotherapy combined with intravitreal anti-VEGF therapy in eyes with exudative AMD.

### 5.7. Gene Therapy

Gene therapy in ophthalmology has garnered renewed interest in recent years since the 2017 FDA approval of voretigene neparvovec-rzyl (Luxturna), a subretinal gene therapy using the AAV2 virus vector to deliver human RPE65 cDNA in RPE65-associated retinal dystrophy [138]. Gene therapy is a novel approach to delivering anti-VEGF therapy long term.

One gene therapy under clinical trial is ixoberogene soroparvovec (formerly ADVM-022) developed by Adverum Biotechnologies (Redwood City, CA, USA). It is undergoing clinical investigation in the phase II LUNA trial for exudative AMD [139]. This therapy uses an AAV.7m8 vector capsid carrying the aflibercept coding sequence on a proprietary expression cassette that is injected intravitreally. Early data of the phase I OPTIC study of six patients who received the higher dose of ADVM-022 have shown maintenance of vision and anatomy without rescue injection for a median of 34 weeks [140,141,142].

Another therapy under investigation for exudative AMD is RGX-314 developed by Regenxbio (Rockville, MD, USA), which has an AAV8 vector that expresses a ranibizumab-like anti-VEGF antibody fragment. It is undergoing clinical trials for injection either into the subretinal or suprachoroidal space [140].

## 6. Conclusions

VEGF has been identified as a key mediator in the pathogenesis of retinal and choroidal neovascularization as well as ME associated with common retinal disorders such as AMD, RVO, and DR. The advent of intravitreal anti-VEGF therapy has been life-changing and vision-saving for many patients with these retinal conditions. Intravitreal delivery of anti-VEGF drugs allows for optimal bioavailability of the drug in the target tissue for maximum therapeutic effect while minimizing potential systemic side effects of anti-VEGF therapy. However, current therapies are expensive and have a limited duration of effect, requiring frequent repeat drug administration.

Fortunately, new advances in drug development are underway to increase the efficacy and durability of intravitreal anti-VEGF therapy and reduce costs. Anti-VEGF biosimilars have been introduced to the market to reduce the cost of intravitreal anti-VEGF therapy. Novel approaches such as gene therapy are being explored in clinical trials and will likely lead to exciting developments in the near future.

## Figures and Tables

**Table 1:** Anti-VEGF therapies.

Drug	Structure	Target	Standard Dose	FDA-Approved Clinical Indications	Landmark Clinical Trials	FDA Approval Date
Pegaptanib	28 nucleotide RNA aptamer with a 5’-attachment of polyethylene glycol	VEGF-A_165_	0.3 mg/0.09 mL	AMD	*AMD*: VISION [62]	2004
Bevacizumab	Recombinant humanized monoclonal IgG1 antibody	All isoforms of VEGF-A	1.25 mg/0.05 mL	Off-label for ophthalmic use	*AMD*: CATT [71] *DME*: BOLT [69], BEVORDEX [70]	Off-label for ophthalmic use
Ranibizumab	Recombinant humanized monoclonal IgG1 kappa antibody fragment	All isoforms of VEGF-A	0.3 mg/0.05 mL or 0.5 mg/0.05 mL	AMD, DR, DME, ME due to RVO, myopic choroidal neovascularization	*AMD*: MARINA [81], ANCHOR [82] *DME*: RISE, RIDE [48] *RVO*: BRAVO, CRUISE [51]	2006
Aflibercept	Soluble decoy receptor comprised of an Fc fragment of IgG1 fused to binding domains of VEGFR-1 and VEGFR-2	All isoforms of VEGF-A, VEGF-B, and placental growth factor	2.0 mg/0.05 mL	AMD, DR, DME, ME due to RVO, retinopathy of prematurity	*AMD*: VIEW 1, VIEW 2 [90] *DME*: VISTA, VIVID [91] *RVO*: COPERNICUS, GALILEO [92]	2011
Brolucizumab	Humanized monoclonal single-chain variable fragment (scFv)	All isoforms of VEGF-A	6.0 mg/0.05 mL	AMD, DME	*AMD*: HAWK, HARRIER [98] *DME*: KESTREL, KITE [102]	2019
Faricimab	IgG1-derived bispecific monoclonal antibody	Angiopoietin-2 and all isoforms of VEGF-A	6.0 mg/0.05 mL	AMD, DME	*AMD*: TENAYA, LUCERNE [108] *DME*: RHINE, YOSEMITE [109]	2022

## Data Availability

Data sharing not applicable. No new data were created or analyzed in this study. Data sharing is not applicable to this article.

## Abbreviations

AMD	Age-related macular degeneration
Anti-VEGF	Drugs that inhibit vascular endothelial growth factor
BRVO	Branch retinal vein occlusion
CRVO	Central retinal vein occlusion
DARPins	Designed ankyrin repeat proteins
DME	diabetic macular edema
DR	Diabetic retinopathy
EG-VEGF	Endocrine gland-derived vascular endothelial growth factor
IOI	Intraocular inflammation
IOP	Intraocular pressure
ME	Macular edema
NP-1	Neuropilin-1
NP-2	Neurophilin-2
NPDR	Non-proliferative diabetic retinopathy
PCV	Polypoidal choroidal vasculopathy
PDR	Proliferative diabetic retinopathy
PDT	Photodynamic therapy
PlGF	Placental growth factor
POHS	Presumed ocular histoplasmosis syndrome
PRP	Panretinal laser photocoagulation
RPE	Retinal pigment epithelium
RVO	Retinal vein occlusion
VEGF	Vascular endothelial growth factor
VEGFR	Vascular endothelial growth factor receptor

## References

[1] Ide A. (1939). Vascularization of the Brown-Pearce rabbit epithelioma transplant as seen in the transparent ear chamber. Am. J. Roentgenol..

[2] Ferrara N. (2011). From the discovery of vascular endothelial growth factor to the introduction of avastin in clinical trials—An interview with Napoleone Ferrara by Domenico Ribatti. Int. J. Dev. Biol..

[3] Melincovici C.S., Boşca A.B., Şuşman S., Mărginean M., Mihu C., Istrate M., Moldovan I.M., Roman A.L., Mihu C.M. (2018). Vascular endothelial growth factor (VEGF)—Key factor in normal and pathological angiogenesis. Rom. J. Morphol. Embryol..

[4] Shibuya M. (2011). Vascular Endothelial Growth Factor (VEGF) and Its Receptor (VEGFR) Signaling in Angiogenesis: A Crucial Target for Anti- and Pro-Angiogenic Therapies. Genes Cancer.

[5] Yonekura H., Sakurai S., Liu X., Migita H., Wang H., Yamagishi S., Nomura M., Abedin M.J., Unoki H., Yamamoto Y. (1999). Placenta growth factor and vascular endothelial growth factor B and C expression in microvascular endothelial cells and pericytes. Implication in autocrine and paracrine regulation of angiogenesis. J. Biol. Chem..

[6] Ferrara N. (2009). VEGF-A: A critical regulator of blood vessel growth. Eur. Cytokine Netw..

[7] Ferrara N., Gerber H.P., LeCouter J. (2003). The biology of VEGF and its receptors. Nat. Med..

[8] Penn J.S., Madan A., Caldwell R.B., Bartoli M., Caldwell R.W., Hartnett M.E. (2008). Vascular endothelial growth factor in eye disease. Prog. Retin. Eye Res..

[9] Bhisitkul R.B. (2006). Vascular endothelial growth factor biology: Clinical implications for ocular treatments. Br. J. Ophthalmol..

[10] Senger D.R., Galli S.J., Dvorak A.M., Perruzzi C.A., Harvey V.S., Dvorak H.F. (1983). Tumor cells secrete a vascular permeability factor that promotes accumulation of ascites fluid. Science.

[11] Leung D.W., Cachianes G., Kuang W.J., Goeddel D.V., Ferrara N. (1989). Vascular endothelial growth factor is a secreted angiogenic mitogen. Science.

[12] Ferrara N., Henzel W.J. (1989). Pituitary follicular cells secrete a novel heparin-binding growth factor specific for vascular endothelial cells. Biochem. Biophys. Res. Commun..

[13] Miyamoto K., Khosrof S., Bursell S.E., Moromizato Y., Aiello L.P., Ogura Y., Adamis A.P. (2000). Vascular endothelial growth factor (VEGF)-induced retinal vascular permeability is mediated by intercellular adhesion molecule-1 (ICAM-1). Am. J. Pathol..

[14] Azzouz M., Ralph G.S., Storkebaum E., Walmsley L.E., Mitrophanous K.A., Kingsman S.M., Carmeliet P., Mazarakis N.D. (2004). VEGF delivery with retrogradely transported lentivector prolongs survival in a mouse ALS model. Nature.

[15] Ding X.M., Mao B.Y., Jiang S., Li S.F., Deng Y.L. (2005). Neuroprotective effect of exogenous vascular endothelial growth factor on rat spinal cord neurons in vitro hypoxia. Chin. Med. J..

[16] Marneros A.G., Fan J., Yokoyama Y., Gerber H.P., Ferrara N., Crouch R.K., Olsen B.R. (2005). Vascular endothelial growth factor expression in the retinal pigment epithelium is essential for choriocapillaris development and visual function. Am. J. Pathol..

[17] Martin A.C., Thornton J.D., Liu J., Wang X., Zuo J., Jablonski M.M., Chaum E., Zindy F., Skapek S.X. (2004). Pathogenesis of persistent hyperplastic primary vitreous in mice lacking the arf tumor suppressor gene. Investig. Ophthalmol. Vis. Sci..

[18] Spaide R.F., Curcio C.A. (2010). Drusen characterization with multimodal imaging. Retina.

[19] Yang S., Zhou J., Li D. (2021). Functions and Diseases of the Retinal Pigment Epithelium. Front. Pharmacol..

[20] Tagawa M., Ooto S., Yamashiro K., Tamura H., Oishi A., Miyata M., Hata M., Yoshikawa M., Yoshimura N., Tsujikawa A. (2020). Characteristics of pachychoroid neovasculopathy. Sci. Rep..

[21] Yamashiro K., Yanagi Y., Koizumi H., Matsumoto H., Cheung C.M.G., Gomi F., Iida T., Tsujikawa A. (2022). Relationship between Pachychoroid and Polypoidal Choroidal Vasculopathy. J. Clin. Med..

[22] Cheung C.M.G., Lai T.Y.Y., Ruamviboonsuk P., Chen S.J., Chen Y., Freund K.B., Gomi F., Koh A.H., Lee W.K., Wong T.Y. (2018). Polypoidal Choroidal Vasculopathy: Definition, Pathogenesis, Diagnosis, and Management. Ophthalmology.

[23] Koh A., Lee W.K., Chen L.J., Chen S.J., Hashad Y., Kim H., Lai T.Y., Pilz S., Ruamviboonsuk P., Tokaji E. (2012). EVEREST study: Efficacy and safety of verteporfin photodynamic therapy in combination with ranibizumab or alone versus ranibizumab monotherapy in patients with symptomatic macular polypoidal choroidal vasculopathy. Retina.

[24] Lim T.H., Lai T.Y.Y., Takahashi K., Wong T.Y., Chen L.J., Ruamviboonsuk P., Tan C.S., Lee W.K., Cheung C.M.G., Ngah N.F. (2020). Comparison of Ranibizumab With or Without Verteporfin Photodynamic Therapy for Polypoidal Choroidal Vasculopathy: The EVEREST II Randomized Clinical Trial. JAMA Ophthalmol..

[25] Oishi A., Miyamoto N., Mandai M., Honda S., Matsuoka T., Oh H., Kita M., Nagai T., Bessho N., Uenishi M. (2014). LAPTOP study: A 24-month trial of verteporfin versus ranibizumab for polypoidal choroidal vasculopathy. Ophthalmology.

[26] Lee W.K., Iida T., Ogura Y., Chen S.J., Wong T.Y., Mitchell P., Cheung G.C.M., Zhang Z., Leal S., Ishibashi T. (2018). Efficacy and Safety of Intravitreal Aflibercept for Polypoidal Choroidal Vasculopathy in the PLANET Study: A Randomized Clinical Trial. JAMA Ophthalmol..

[27] Kokame G.T., Lai J.C., Wee R., Yanagihara R., Shantha J.G., Ayabe J., Hirai K. (2016). Prospective clinical trial of Intravitreal aflibercept treatment for PolypoIdal choroidal vasculopathy with hemorrhage or exudation (EPIC study): 6 month results. BMC Ophthalmol..

[28] Seko Y., Seko Y., Fujikura H., Pang J., Tokoro T., Shimokawa H. (1999). Induction of vascular endothelial growth factor after application of mechanical stress to retinal pigment epithelium of the rat in vitro. Investig. Ophthalmol. Vis. Sci..

[29] Tan C.S., Sadda S.R. (2017). Anti-vascular endothelial growth factor therapy for the treatment of myopic choroidal neovascularization. Clin. Ophthalmol..

[30] Barth T., Zeman F., Helbig H., Gamulescu M.A. (2019). Intravitreal anti-VEGF treatment for choroidal neovascularization secondary to traumatic choroidal rupture. BMC Ophthalmol..

[31] Wong T.Y., Sun J., Kawasaki R., Ruamviboonsuk P., Gupta N., Lansingh V.C., Maia M., Mathenge W., Moreker S., Muqit M.M.K. (2018). Guidelines on Diabetic Eye Care: The International Council of Ophthalmology Recommendations for Screening, Follow-up, Referral, and Treatment Based on Resource Settings. Ophthalmology.

[32] Evans J.R., Michelessi M., Virgili G. (2014). Laser photocoagulation for proliferative diabetic retinopathy. Cochrane Database Syst. Rev..

[33] Gross J.G., Glassman A.R., Liu D., Sun J.K., Antoszyk A.N., Baker C.W., Bressler N.M., Elman M.J., Ferris F.L., Gardner T.W. (2018). Five-Year Outcomes of Panretinal Photocoagulation vs Intravitreous Ranibizumab for Proliferative Diabetic Retinopathy: A Randomized Clinical Trial. JAMA Ophthalmol..

[34] Maturi R.K., Glassman A.R., Josic K., Antoszyk A.N., Blodi B.A., Jampol L.M., Marcus D.M., Martin D.F., Melia M., Salehi-Had H. (2021). Effect of Intravitreous Anti-Vascular Endothelial Growth Factor vs Sham Treatment for Prevention of Vision-Threatening Complications of Diabetic Retinopathy: The Protocol W Randomized Clinical Trial. JAMA Ophthalmol..

[35] Maturi R.K., Glassman A.R., Josic K., Baker C.W., Gerstenblith A.T., Jampol L.M., Meleth A., Martin D.F., Melia M., Punjabi O.S. (2023). Four-Year Visual Outcomes in the Protocol W Randomized Trial of Intravitreous Aflibercept for Prevention of Vision-Threatening Complications of Diabetic Retinopathy. JAMA.

[36] Nicholson L., Talks S.J., Amoaku W., Talks K., Sivaprasad S. (2022). Retinal vein occlusion (RVO) guideline: Executive summary. Eye.

[37] The Eye Disease Case-Control Study Group (1996). Risk Factors for Central Retinal Vein Occlusion. Arch. Ophthalmol..

[38] Karia N. (2010). Retinal vein occlusion: Pathophysiology and treatment options. Clin. Ophthalmol..

[39] Garweg J.G., Zandi S. (2016). Retinal vein occlusion and the use of a dexamethasone intravitreal implant (Ozurdex^®^) in its treatment. Graefes Arch. Clin. Exp. Ophthalmol..

[40] Heier J.S., Campochiaro P.A., Yau L., Li Z., Saroj N., Rubio R.G., Lai P. (2012). Ranibizumab for macular edema due to retinal vein occlusions: Long-term follow-up in the HORIZON trial. Ophthalmology.

[41] Brown D.M., Campochiaro P.A., Singh R.P., Li Z., Gray S., Saroj N., Rundle A.C., Rubio R.G., Murahashi W.Y. (2010). Ranibizumab for macular edema following central retinal vein occlusion: Six-month primary end point results of a phase III study. Ophthalmology.

[42] Senthil S., Dada T., Das T., Kaushik S., Puthuran G.V., Philip R., Rani P.K., Rao H., Singla S., Vijaya L. (2021). Neovascular glaucoma—A review. Indian J. Ophthalmol..

[43] Bhatnagar A., Skrehot H.C., Bhatt A., Herce H., Weng C.Y. (2023). Epidemiology of Retinopathy of Prematurity in the US From 2003 to 2019. JAMA Ophthalmol..

[44] Hellström A., Smith L.E., Dammann O. (2013). Retinopathy of prematurity. Lancet.

[45] Mintz-Hittner H.A., Kennedy K.A., Chuang A.Z. (2011). Efficacy of intravitreal bevacizumab for stage 3+ retinopathy of prematurity. N. Engl. J. Med..

[46] Tran K.D., Cernichiaro-Espinosa L.A., Berrocal A.M. (2018). Management of Retinopathy of Prematurity—Use of Anti-VEGF Therapy. Asia Pac. J. Ophthalmol..

[47] Brownlee M. (2005). The pathobiology of diabetic complications: A unifying mechanism. Diabetes.

[48] Nguyen Q.D., Brown D.M., Marcus D.M., Boyer D.S., Patel S., Feiner L., Gibson A., Sy J., Rundle A.C., Hopkins J.J. (2012). Ranibizumab for diabetic macular edema: Results from 2 phase III randomized trials: RISE and RIDE. Ophthalmology.

[49] Wells J.A., Glassman A.R., Ayala A.R., Jampol L.M., Aiello L.P., Antoszyk A.N., Arnold-Bush B., Baker C.W., Bressler N.M., Browning D.J. (2015). Aflibercept, bevacizumab, or ranibizumab for diabetic macular edema. N. Engl. J. Med..

[50] Noma H., Yasuda K., Shimura M. (2020). Cytokines and Pathogenesis of Central Retinal Vein Occlusion. J. Clin. Med..

[51] Varma R., Bressler N.M., Suñer I., Lee P., Dolan C.M., Ward J., Colman S., Rubio R.G. (2012). Improved vision-related function after ranibizumab for macular edema after retinal vein occlusion: Results from the BRAVO and CRUISE trials. Ophthalmology.

[52] Heier J.S., Clark W.L., Boyer D.S., Brown D.M., Vitti R., Berliner A.J., Kazmi H., Ma Y., Stemper B., Zeitz O. (2014). Intravitreal aflibercept injection for macular edema due to central retinal vein occlusion: Two-year results from the COPERNICUS study. Ophthalmology.

[53] Clark W.L., Boyer D.S., Heier J.S., Brown D.M., Haller J.A., Vitti R., Kazmi H., Berliner A.J., Erickson K., Chu K.W. (2016). Intravitreal Aflibercept for Macular Edema Following Branch Retinal Vein Occlusion: 52-Week Results of the VIBRANT Study. Ophthalmology.

[54] Archer D.B., Amoaku W.M., Gardiner T.A. (1991). Radiation retinopathy--clinical, histopathological, ultrastructural and experimental correlations. Eye.

[55] Mason J.O., Albert M.A., Persaud T.O., Vail R.S. (2007). Intravitreal bevacizumab treatment for radiation macular edema after plaque radiotherapy for choroidal melanoma. Retina.

[56] Rabb M.F., Gagliano D.A., Teske M.P. (1988). Retinal arterial macroaneurysms. Surv. Ophthalmol..

[57] Chen Y.Y., Lin L.Y., Chang P.Y., Chen F.T., Mai E.L.C., Wang J.K. (2017). Laser and Anti-Vascular Endothelial Growth Factor Agent Treatments for Retinal Arterial Macroaneurysm. Asia Pac. J. Ophthalmol..

[58] Takkar B., Roy S., Sodhi P.K., Azad S., Bajwa G.S. (2017). Peripheral choroidal neovascular membrane in a case of peripheral exudative hemorrhagic chorioretinopathy managed with combination therapy. Int. Ophthalmol..

[59] Vinores S.A. (2006). Pegaptanib in the treatment of wet, age-related macular degeneration. Int. J. Nanomed..

[60] (2002). Preclinical and phase 1A clinical evaluation of an anti-VEGF pegylated aptamer (EYE001) for the treatment of exudative age-related macular degeneration. Retina.

[61] Patel M., Whitfield L., Hutmacher M., Kowalski K., Burger P., Dessalegn B., Modi M., Group M.S. (2006). Population Pharmacokinetics/Pharmacodynamics (PK/PD) of Pegaptanib Sodium (Macugen^®^) in Patients with Age–Related Macular Degeneration (AMD). Investig. Ophthalmol. Vis. Sci..

[62] Gragoudas E.S., Adamis A.P., Cunningham E.T., Jr Feinsod M., Guyer D.R. (2004). Pegaptanib for neovascular age-related macular degeneration. N. Engl. J. Med..

[63] Stewart M.W. (2012). Vascular Endothelial Growth Factor Inhibitor Use in Ophthalmology: The Revolution Continues. Curr. Drug Ther..

[64] Ferrara N., Hillan K.J., Gerber H.P., Novotny W. (2004). Discovery and development of bevacizumab, an anti-VEGF antibody for treating cancer. Nat. Rev. Drug Discov..

[65] Grisanti S., Ziemssen F. (2007). Bevacizumab: Off-label use in ophthalmology. Indian J. Ophthalmol..

[66] Michels S., Rosenfeld P.J., Puliafito C.A., Marcus E.N., Venkatraman A.S. (2005). Systemic bevacizumab (Avastin) therapy for neovascular age-related macular degeneration twelve-week results of an uncontrolled open-label clinical study. Ophthalmology.

[67] Rosenfeld P.J., Fung A.E., Puliafito C.A. (2005). Optical coherence tomography findings after an intravitreal injection of bevacizumab (avastin) for macular edema from central retinal vein occlusion. Ophthalmic Surg. Lasers Imaging.

[68] Rosenfeld P.J., Moshfeghi A.A., Puliafito C.A. (2005). Optical coherence tomography findings after an intravitreal injection of bevacizumab (avastin) for neovascular age-related macular degeneration. Ophthalmic Surg. Lasers Imaging.

[69] Michaelides M., Kaines A., Hamilton R.D., Fraser-Bell S., Rajendram R., Quhill F., Boos C.J., Xing W., Egan C., Peto T. (2010). A prospective randomized trial of intravitreal bevacizumab or laser therapy in the management of diabetic macular edema (BOLT study) 12-month data: Report 2. Ophthalmology.

[70] Gillies M.C., Lim L.L., Campain A., Quin G.J., Salem W., Li J., Goodwin S., Aroney C., McAllister I.L., Fraser-Bell S. (2014). A randomized clinical trial of intravitreal bevacizumab versus intravitreal dexamethasone for diabetic macular edema: The BEVORDEX study. Ophthalmology.

[71] Martin D.F., Maguire M.G., Ying G.S., Grunwald J.E., Fine S.L., Jaffe G.J. (2011). Ranibizumab and bevacizumab for neovascular age-related macular degeneration. N. Engl. J. Med..

[72] Afarid M., Sadegi Sarvestani A., Rahat F., Azimi A. (2018). Intravitreal Injection of Bevacizumab: Review of our previous Experience. Iran. J. Pharm. Res..

[73] Modarreszadeh M., Naseripour M., Ghasemi-Falavarjani K., Nikeghbali A., Hashemi M., Parvaresh M.M. (2008). Two Different Doses of Intravitreal Bevacizumab for Treatment of Choroidal Neovascularization Associated with Age-related Macular Degeneration. J. Ophthalmic Vis. Res..

[74] Wu L., Arevalo J.F., Roca J.A., Maia M., Berrocal M.H., Rodriguez F.J., Evans T., Costa R.A., Cardillo J. (2008). Comparison of two doses of intravitreal bevacizumab (Avastin) for treatment of macular edema secondary to branch retinal vein occlusion: Results from the Pan-American Collaborative Retina Study Group at 6 months of follow-up. Retina.

[75] Jan S., Nazim M., Karim S., Hussain Z. (2016). Intravitreal Bevacizumab: Indications And Complications. J. Ayub Med. Coll. Abbottabad.

[76] Fasih U., Shaikh N., Rahman A., Sultan S., Fehmi M.S., Shaikh A. (2013). A one-year follow-up study of ocular and systemic complications of intravitreal injection of bevacizumab (Avastin). J. Pak. Med. Assoc..

[77] VanderBeek B.L., Bonaffini S.G., Ma L. (2015). Association of compounded bevacizumab with postinjection endophthalmitis. JAMA Ophthalmol..

[78] Kim L.A., D’Amore P.A. (2012). A brief history of anti-VEGF for the treatment of ocular angiogenesis. Am. J. Pathol..

[79] Lien S., Lowman H.B. (2008). Therapeutic anti-VEGF antibodies. Handb. Exp. Pharmacol..

[80] Joo K., Park S.J., Choi Y., Lee J.E., Na Y.M., Hong H.K., Park K.H., Kim H.M., Chung J.Y., Woo S.J. (2017). Role of the Fc Region in the Vitreous Half-Life of Anti-VEGF Drugs. Investig. Ophthalmol. Vis. Sci..

[81] Rosenfeld P.J., Brown D.M., Heier J.S., Boyer D.S., Kaiser P.K., Chung C.Y., Kim R.Y. (2006). Ranibizumab for neovascular age-related macular degeneration. N. Engl. J. Med..

[82] Brown D.M., Kaiser P.K., Michels M., Soubrane G., Heier J.S., Kim R.Y., Sy J.P., Schneider S. (2006). Ranibizumab versus verteporfin for neovascular age-related macular degeneration. N. Engl. J. Med..

[83] Boyer D.S., Heier J.S., Brown D.M., Francom S.F., Ianchulev T., Rubio R.G. (2009). A Phase IIIb study to evaluate the safety of ranibizumab in subjects with neovascular age-related macular degeneration. Ophthalmology.

[84] Vaidyanathan U., Moshirfar M. (2023). Ranibizumab. StatPearls.

[85] Adams B.S., Sorhaitz W., Stringham J. (2023). Aflibercept. StatPearls.

[86] Papadopoulos N., Martin J., Ruan Q., Rafique A., Rosconi M.P., Shi E., Pyles E.A., Yancopoulos G.D., Stahl N., Wiegand S.J. (2012). Binding and neutralization of vascular endothelial growth factor (VEGF) and related ligands by VEGF Trap, ranibizumab and bevacizumab. Angiogenesis.

[87] Stewart M.W., Rosenfeld P.J. (2008). Predicted biological activity of intravitreal VEGF Trap. Br. J. Ophthalmol..

[88] Autiero M., Waltenberger J., Communi D., Kranz A., Moons L., Lambrechts D., Kroll J., Plaisance S., De Mol M., Bono F. (2003). Role of PlGF in the intra- and intermolecular cross talk between the VEGF receptors Flt1 and Flk1. Nat. Med..

[89] Heier J.S., Brown D.M., Chong V., Korobelnik J.F., Kaiser P.K., Nguyen Q.D., Kirchhof B., Ho A., Ogura Y., Yancopoulos G.D. (2012). Intravitreal aflibercept (VEGF trap-eye) in wet age-related macular degeneration. Ophthalmology.

[90] Schmidt-Erfurth U., Kaiser P.K., Korobelnik J.F., Brown D.M., Chong V., Nguyen Q.D., Ho A.C., Ogura Y., Simader C., Jaffe G.J. (2014). Intravitreal aflibercept injection for neovascular age-related macular degeneration: Ninety-six-week results of the VIEW studies. Ophthalmology.

[91] Heier J.S., Korobelnik J.F., Brown D.M., Schmidt-Erfurth U., Do D.V., Midena E., Boyer D.S., Terasaki H., Kaiser P.K., Marcus D.M. (2016). Intravitreal Aflibercept for Diabetic Macular Edema: 148-Week Results from the VISTA and VIVID Studies. Ophthalmology.

[92] Pielen A., Clark W.L., Boyer D.S., Ogura Y., Holz F.G., Korobelnik J.F., Stemper B., Asmus F., Rittenhouse K.D., Ahlers C. (2017). Integrated results from the COPERNICUS and GALILEO studies. Clin. Ophthalmol..

[93] Wells J.A., Glassman A.R., Ayala A.R., Jampol L.M., Bressler N.M., Bressler S.B., Brucker A.J., Ferris F.L., Hampton G.R., Jhaveri C. (2016). Aflibercept, Bevacizumab, or Ranibizumab for Diabetic Macular Edema: Two-Year Results from a Comparative Effectiveness Randomized Clinical Trial. Ophthalmology.

[94] Wykoff C.C., Brown D.M., Reed K., Berliner A.J., Gerstenblith A.T., Breazna A., Abraham P., Fein J.G., Chu K.W., Clark W.L. Effect of High-Dose Intravitreal Aflibercept, 8 mg, in Patients With Neovascular Age-Related Macular Degeneration: The Phase 2 CANDELA Randomized Clinical Trial. JAMA Ophthalmol..

[95] Iyer P.G., Albini T.A. (2021). Drug-related adverse effects of antivascular endothelial growth factor agents. Curr. Opin. Ophthalmol..

[96] Markham A. (2019). Brolucizumab: First Approval. Drugs.

[97] Gaudreault J., Gunde T., Floyd H.S., Ellis J., Tietz J., Binggeli D., Keller B., Schmidt A., Escher D. (2012). Preclinical pharmacology and safety of ESBA1008, a single-chain antibody fragment, investigated as potential treatment for age related macular degeneration. Investig. Ophthalmol. Vis. Sci..

[98] Dugel P.U., Koh A., Ogura Y., Jaffe G.J., Schmidt-Erfurth U., Brown D.M., Gomes A.V., Warburton J., Weichselberger A., Holz F.G. (2020). HAWK and HARRIER: Phase 3, Multicenter, Randomized, Double-Masked Trials of Brolucizumab for Neovascular Age-Related Macular Degeneration. Ophthalmology.

[99] Tietz J., Spohn G., Schmid G., Konrad J., Jampen S., Maurer P., Schmidt A., Escher D. (2015). Affinity and potency of RTH258 (ESBA1008), a novel inhibitor of vascular endothelial growth factor a for the treatment of retinal disorders. Investig. Ophthalmol. Vis. Sci..

[100] Dugel P.U., Singh R.P., Koh A., Ogura Y., Weissgerber G., Gedif K., Jaffe G.J., Tadayoni R., Schmidt-Erfurth U., Holz F.G. (2021). HAWK and HARRIER: Ninety-Six-Week Outcomes from the Phase 3 Trials of Brolucizumab for Neovascular Age-Related Macular Degeneration. Ophthalmology.

[101] Mehta N., Fong R.D., Wilson M., Moussa K., Emami-Naeini P., Moshiri A., Yiu G., Park S.S. (2023). Real-World Experience Using Intravitreal Brolucizumab Alone or in Combination with Aflibercept in the Management of Neovascular Age-Related Macular Degeneration. Clin. Ophthalmol..

[102] Brown D.M., Emanuelli A., Bandello F., Barranco J.J.E., Figueira J., Souied E., Wolf S., Gupta V., Ngah N.F., Liew G. (2022). KESTREL and KITE: 52-Week Results From Two Phase III Pivotal Trials of Brolucizumab for Diabetic Macular Edema. Am. J. Ophthalmol..

[103] Baumal C.R., Spaide R.F., Vajzovic L., Freund K.B., Walter S.D., John V., Rich R., Chaudhry N., Lakhanpal R.R., Oellers P.R. (2020). Retinal Vasculitis and Intraocular Inflammation after Intravitreal Injection of Brolucizumab. Ophthalmology.

[104] Jain A., Chea S., Matsumiya W., Halim M.S., Yaşar Ç., Kuang G., Sepah Y.J., Khanani A.M., Do D.V., Nguyen Q.D. (2020). Severe vision loss secondary to retinal arteriolar occlusions after multiple intravitreal brolucizumab administrations. Am. J. Ophthalmol. Case Rep..

[105] Singer M., Albini T.A., Seres A., Baumal C.R., Parikh S., Gale R., Kaiser P.K., Lobach I., Feltgen N., Joshi M.R. (2022). Clinical Characteristics and Outcomes of Eyes with Intraocular Inflammation after Brolucizumab: Post Hoc Analysis of HAWK and HARRIER. Ophthalmol. Retin..

[106] Shirley M. (2022). Faricimab: First Approval. Drugs.

[107] Khanani A.M., Russell M.W., Aziz A.A., Danzig C.J., Weng C.Y., Eichenbaum D.A., Singh R.P. (2021). Angiopoietins as Potential Targets in Management of Retinal Disease. Clin. Ophthalmol..

[108] Heier J.S., Khanani A.M., Quezada Ruiz C., Basu K., Ferrone P.J., Brittain C., Figueroa M.S., Lin H., Holz F.G., Patel V. (2022). Efficacy, durability, and safety of intravitreal faricimab up to every 16 weeks for neovascular age-related macular degeneration (TENAYA and LUCERNE): Two randomised, double-masked, phase 3, non-inferiority trials. Lancet.

[109] Wykoff C.C., Abreu F., Adamis A.P., Basu K., Eichenbaum D.A., Haskova Z., Lin H., Loewenstein A., Mohan S., Pearce I.A. (2022). Efficacy, durability, and safety of intravitreal faricimab with extended dosing up to every 16 weeks in patients with diabetic macular oedema (YOSEMITE and RHINE): Two randomised, double-masked, phase 3 trials. Lancet.

[110] Rasier R., Artunay O., Yuzbasioglu E., Sengul A., Bahcecioglu H. (2009). The effect of intravitreal bevacizumab (avastin) administration on systemic hypertension. Eye.

[111] Sengul A., Rasier R., Ciftci C., Artunay O., Kockar A., Bahcecioglu H., Yuzbasioglu E. (2017). Short-term effects of intravitreal ranibizumab and bevacizumab administration on 24-h ambulatory blood pressure monitoring recordings in normotensive patients with age-related macular degeneration. Eye.

[112] Thulliez M., Angoulvant D., Le Lez M.L., Jonville-Bera A.P., Pisella P.J., Gueyffier F., Bejan-Angoulvant T. (2014). Cardiovascular events and bleeding risk associated with intravitreal antivascular endothelial growth factor monoclonal antibodies: Systematic review and meta-analysis. JAMA Ophthalmol..

[113] Holekamp N.M., Campochiaro P.A., Chang M.A., Miller D., Pieramici D., Adamis A.P., Brittain C., Evans E., Kaufman D., Maass K.F. (2022). Archway randomized phase 3 trial of the port delivery system with ranibizumab for neovascular age-related macular degeneration. Ophthalmology.

[114] Regillo C., Berger B., Brooks L., Clark W.L., Mittra R., Wykoff C.C., Callaway N.F., DeGraaf S., Ding H.T., Fung A.E. (2023). Archway Phase 3 Trial of the Port Delivery System with Ranibizumab for Neovascular Age-Related Macular Degeneration 2-Year Results. Ophthalmology.

[115] Sharma A., Khanani A.M., Parachuri N., Kumar N., Bandello F., Kuppermann B.D. (2023). Port delivery system with ranibizumab (Susvimo) recall- What does it mean to the retina specialists. Int. J. Retina Vitr..

[116] American Academy of Ophthalmology, Academy Board of Trustees The Use of Biosimilars in Ophthalmic Practice—2022. https://www.aao.org/education/clinical-statement/use-of-biosimilars-in-ophthalmic-practice.

[117] Sharma A., Kumar N., Kuppermann B.D., Francesco B., Lowenstein A. (2019). Ophthalmic biosimilars: Lessons from India. Indian J. Ophthalmol..

[118] Sharma A., Kumar N., Kuppermann B.D., Bandello F., Loewenstein A. (2020). Ophthalmic biosimilars and biologics-role of endotoxins. Eye.

[119] Kaiser P.K., Schmitz-Valckenberg M.S., Holz F.G. (2022). Anti-Vascular Endothelial Growth Factor Biosimilars in Ophthalmology. Retina.

[120] Comparing the Efficacy and Safety of Biosimilar Candidate Xlucane Versus Lucentis® in Patients with nAMD (XPLORE). https://classic.clinicaltrials.gov/ct2/show/NCT03805100.

[121] Kapur M., Nirula S., Naik M.P. (2022). Future of anti-VEGF: Biosimilars and biobetters. Int. J. Retina Vitreous.

[122] Outlook Therapeutics Presents NORSE TWO Phase 3 Pivotal Safety and Efficacy Data for ONS-5010/LYTENAVA™ (Bevacizumab-Vikg) at the Retina Subspecialty Day, American Academy of Ophthalmology (AAO) 2021 Annual Conference. https://ir.outlooktherapeutics.com/news-releases/news-release-details/outlook-therapeutics-presents-norse-two-phase-3-pivotal-safety.

[123] Stumpp M.T., Binz H.K., Amstutz P. (2008). DARPins: A new generation of protein therapeutics. Drug Discov. Today.

[124] Khurana R.N., Kunimoto D., Yoon Y.H., Wykoff C.C., Chang A., Maturi R.K., Agostini H., Souied E., Chow D.R., Lotery A.J. (2021). Two-Year Results of the Phase 3 Randomized Controlled Study of Abicipar in Neovascular Age-Related Macular Degeneration. Ophthalmology.

[125] Kunimoto D., Yoon Y.H., Wykoff C.C., Chang A., Khurana R.N., Maturi R.K., Agostini H., Souied E., Chow D.R., Lotery A.J. (2020). Efficacy and Safety of Abicipar in Neovascular Age-Related Macular Degeneration: 52-Week Results of Phase 3 Randomized Controlled Study. Ophthalmology.

[126] Sharma A., Kumar N., Kuppermann B.D., Bandello F. (2020). Abicipar pegol: The non-monoclonal antibody anti-VEGF. Eye.

[127] Callanan D., Khurana R.N., Maturi R.K., Patel S., Wykoff C.C., Eichenbaum D., Khanani A.M., Hassan T., Badger H., Mehta S. (2023). Impact of Modifying Abicipar Manufacturing Process in Patients with Neovascular Age-Related Macular Degeneration: MAPLE Study Results. Clin. Ophthalmol..

[128] Li J., Mooney D.J. (2016). Designing hydrogels for controlled drug delivery. Nat. Rev. Mater..

[129] Matanović M.R., Kristl J., Grabnar P.A. (2014). Thermoresponsive polymers: Insights into decisive hydrogel characteristics, mechanisms of gelation, and promising biomedical applications. Int. J. Pharm..

[130] Kim S., Kang-Mieler J.J., Liu W., Wang Z., Yiu G., Teixeira L.B.C., Mieler W.F., Thomasy S.M. (2020). Safety and Biocompatibility of Aflibercept-Loaded Microsphere Thermo-Responsive Hydrogel Drug Delivery System in a Nonhuman Primate Model. Transl. Vis. Sci. Technol..

[131] Hussain R.M., Shaukat B.A., Ciulla L.M., Berrocal A.M., Sridhar J. (2021). Vascular Endothelial Growth Factor Antagonists: Promising Players in the Treatment of Neovascular Age-Related Macular Degeneration. Drug Des. Devel Ther..

[132] Le Tourneau C., Raymond E., Faivre S. (2007). Sunitinib: A novel tyrosine kinase inhibitor. A brief review of its therapeutic potential in the treatment of renal carcinoma and gastrointestinal stromal tumors (GIST). Ther. Clin. Risk Manag..

[133] Samanta A., Aziz A.A., Jhingan M., Singh S.R., Khanani A.M., Chhablani J. (2020). Emerging Therapies in Neovascular Age-Related Macular Degeneration in 2020. Asia Pac. J. Ophthalmol..

[134] Tolentino M.J., Tolentino A.J. (2022). Investigational drugs in clinical trials for macular degeneration. Expert. Opin. Investig. Drugs.

[135] Osmanovic S., Moisseiev E., Mishra K.K., Daftari I., Moshiri A., Morse L., Park S.S. (2017). Phase I/II Randomized Study of Proton Beam with Anti-Vascular Endothelial Growth Factor for Exudative Age-Related Macular Degeneration: One-Year Results. Ophthalmol. Retin..

[136] Mukkamala L.K., Mishra K., Daftari I., Moshiri A., Park S.S. (2020). Phase I/II randomized study of proton beam with anti-VEGF for exudative age-related macular degeneration: Long-term results. Eye.

[137] Dugel P.U., Bebchuk J.D., Nau J., Reichel E., Singer M., Barak A., Binder S., Jackson T.L. (2013). Epimacular brachytherapy for neovascular age-related macular degeneration: A randomized, controlled trial (CABERNET). Ophthalmology.

[138] Maguire A.M., Bennett J., Aleman E.M., Leroy B.P., Aleman T.S. (2021). Clinical Perspective: Treating RPE65-Associated Retinal Dystrophy. Mol. Ther..

[139] Adverum Biotechnologies, Inc. Adverum Biotechnologies Announces First Subject Dosed with Ixo-Vec in the Phase 2 LUNA Trial for the Treatment of Wet Age-Related Macular Degeneration. https://investors.adverum.com/news/news-details/2022/Adverum-Biotechnologies-Announces-First-Subject-Dosed-with-Ixo-vec-in-the-Phase-2-LUNA-Trial-for-the-Treatment-of-Wet-Age-Related-Macular-Degeneration/default.aspx.

[140] Chung S.H., Frick S.L., Yiu G. (2021). Targeting vascular endothelial growth factor using retinal gene therapy. Ann. Transl. Med..

[141] Grishanin R., Vuillemenot B., Sharma P., Keravala A., Greengard J., Gelfman C., Blumenkrantz M., Lawrence M., Hu W., Kiss S. (2019). Preclinical Evaluation of ADVM-022, a Novel Gene Therapy Approach to Treating Wet Age-Related Macular Degeneration. Mol. Ther..

[142] Khanani A.M., Kiss S., Turpcu A., Hoang C., Osborne A. (2020). Phase 1 study of intravitreal gene therapy ADVM-022 for neovascular AMD (OPTIC Trial). Investig. Ophthalmol. Vis. Sci..

